# Employing Metadynamics to Predict the Membrane Partitioning of Carboxy-2*H*-Azirine Natural Products

**DOI:** 10.1021/acs.jpcb.4c03411

**Published:** 2024-09-03

**Authors:** Clyde A. Daly, Leah M. Seebald, Emma Wolk

**Affiliations:** Department of Chemistry, Haverford College, 370 Lancaster Ave., Haverford, Pennsylvania 19041, United States

## Abstract

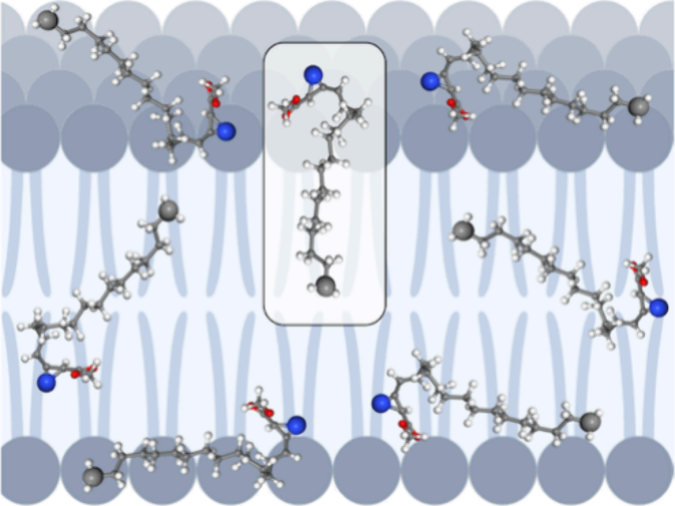

Natural products containing the carboxy-2*H*-azirine moiety are an exciting target for investigation due to their broad-spectrum antimicrobial activity and new chemical space they afford for novel therapeutic development. The carboxy-2*H*-azirine moiety, including those appended to well-characterized chemical scaffolds, is understudied, which creates a challenge for understanding potential modes of inhibition. In particular, some known natural product carboxy-2*H*-azirines have long hydrophobic tails, which could implicate them in membrane-associated processes. In this study, we examined a small set of carboxy-2*H*-azirine natural products with varied structural features that could alter membrane partitioning. We compared the predicted membrane partitioning and alignment of these compounds to those of established membrane embedders with similar chemical scaffolds. To accomplish this, we developed parameters within the framework of the CHARMM36 force field for the 2*H*-azirine functional group and performed metadynamics simulations of the partitioning into a model bacterial membrane from aqueous solution. We determined that the carboxy-2*H*-azirine functional group is strongly hydrophilic, imbuing the long-chain natural products with amphipathicity similar to the known membrane-embedding molecules to which they were compared. For the long-chain analogs, the carboxy-2*H*-azirine head group stays within 1 nm of the phosphate layer, while the hydrophobic tail sits within the membrane. The carboxy-2*H*-azirine lacking the long alkyl chain instead partitions completely into aqueous solution.

## Introduction

The alarming global increase in antimicrobial resistance has created a pressing need to expand the chemical space of antimicrobials to mitigate infectious diseases.^1^ Natural products containing a carboxy-2*H*-azirine moiety have garnered attention for demonstrating moderate inhibition against resistant microbes,^2−8^ which provides a strong starting scaffold that can be optimized through structure–activity relationships (SAR).^3,4,6^ Despite these intriguing inhibition results, the biological target of inhibition is currently unknown. The carboxy-2*H*-azirine is a unique and understudied moiety, and its structure does not readily inspire confident assumptions about physiological behavior. To clarify the properties of this moiety and begin to build an understanding of its antimicrobial activity, we set out to predict the membrane partitioning behavior of molecules with a carboxy-2*H*-azirine functional group in a bacterial lipid bilayer. This includes investigating the orientation of these molecules when embedded in a lipid bilayer, as well as how these molecules protrude in relation to the polar phospholipid head groups of the lipid bilayer.

Structural features of natural products can often supply hints to infer biological targets. For example, many natural products known to inhibit membrane protein activity or disrupt membrane integrated processes have a distinctive amphipathic scaffold.^9^ This correlation between amphipathic structure and inhibition of a membrane target may be hypothesized to exist for the carboxy-2*H*-azirine natural products. Many of these carboxy-2*H*-azirine natural products comprise a long hydrophobic alkyl chain ranging from 16 to 18 carbons, suggestive of integration into a membrane lipid bilayer. On the other hand, the physiochemical properties of just the carboxy-2*H*-azirine moiety are not nearly as well-established as products. While the long alkyl chains of some of these compounds are suggestive of lipid embedding, the influence of the carboxy-2*H*-azirine on these membrane interactions is unclear because the hydrophilicity of the carboxy-2*H*-azirine has not been characterized.

To provide predictive clarity, we first examine structurally similar molecules to help guide predictions of the physicochemical properties of a carboxy-2*H*-azirine interacting with a membrane lipid bilayer. Computational tools such as molecular dynamics (MD) have been used to make predictions of membrane partitioning, permeability, and small-molecule embedding.^11−15^ While extensive force fields for biomolecules and many small molecules exist, there has been little effort to parametrize carboxy-2*H*-azirines. Most computational modeling of this moiety has been performed using electronic structure methods where force fields are not needed.^16−21^ However the processes at question in this work cannot be addressed by electronic structure methods. Some studies have used automatic parameter generation without adjustment to obtain force fields for small azirine-containing molecules.^21−23^ We hope to address the limitations of these studies by avoiding parameter assignment by *pure* analogy since there are few well-characterized and analogous moieties to carboxy-2*H-*azirines.^25,26^

Typical MD simulations can fully characterize the behavior of molecular processes with timescales between 1 ps and 1 μs.^11,12,27^ Because of the slow dynamics of membranes, straightforward atomistic simulations tend not to be able to reliably capture the equilibrium behavior of arbitrary small molecules relative to the bilayer.^11,12,28^ However, enhanced sampling methods can overcome these limitations.^29,30^ In these methods, the probability that configurations are sampled by the simulation is manipulated such that the full space of some collective variable (CV) is observed. Metadynamics can be used to sample a particular CV and reconstruct the potential of mean force (PMF).^31,32^ Here, biases are added to the potential energy surface to encourage sampling of the collective variable; the accumulated biases can be used at the end of the simulation to reconstruct the potential of mean force.^31,32^

We use metadynamics as a tool to quantify the propensity of these carboxy-2*H*-azirine natural products to integrate in representative bacterial lipid bilayers. To accomplish this, we will obtain the PMF for moving the molecules into and out of the membrane. The PMF will allow us to calculate the free energy for membrane partitioning, thus determining if the molecules are thermodynamically stable in the membrane or in the aqueous environment. We also investigate the orientation and solvent exposure of the molecules of interest by unbiasing the statistics from our biased simulations.

**Figure 1:**
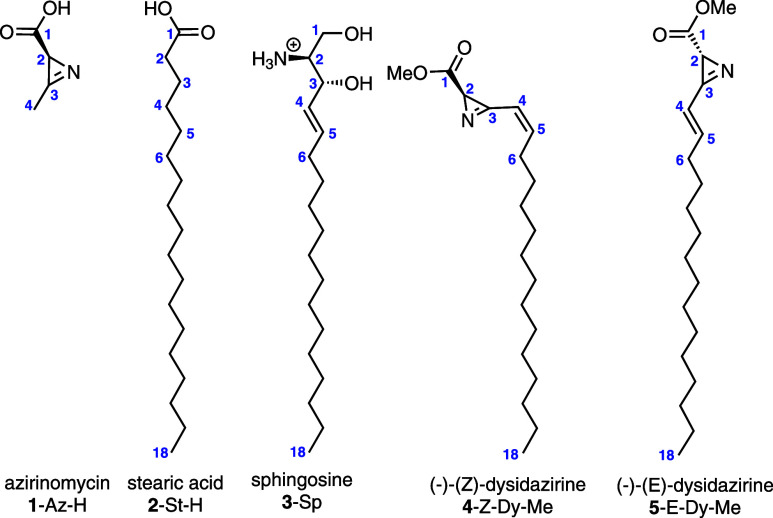
Structures of the molecules investigated in this study. Carbons are numbered in blue according to IUPAC rules. Notable similarities include the alkyl chain length, the C4–C5 unsaturation, and the nitrogen and oxygen heteroatoms at C1 and C2.

These simulations are performed for molecules **1**-Az-H, **4**-Z-Dy-Me, and **5**-E-Dy-Me and two structurally similar molecules known to exhibit membrane embedding: stearic acid (**2**-St-H) and sphingosine (**3**-Sp) (Figure 1). Compounds **2**-St-H, **3**-Sp, **4**-Z-Dy-Me, and **5**-E-Dy-Me all share a linear carbon chain comprising 18 carbons. More specifically, **4**-Z-Dy-Me, **5**-E-Dy-Me, and **3**-Sp are unsaturated between C4 and C5. Regarding this unsaturation, **4**-Z-Dy-Me has the *Z* configuration, providing contrast to **5**-E-Dy-ME and **3**-Sp, which have the *E* configuration. Compound **3**-Sp is saturated between C4 and C5, so this compound was used as a comparative model for membrane partitioning of different linear C18 alkyl chains. If the carboxy-2*H*-azirine group is overall hydrophilic, then we should expect to see similar membrane partitioning and alignment behavior for all four long-chain molecules in our investigated set. The greatest uncertainty in this prediction arises from the difference in oxidation at C1 of **4***-*Z-Dy-Me and **5**-E-Dy-Me compared to **3**-Sp, in combination with the understudied properties of the 2*H*-azirine heterocycle, especially related to hydrophilicity. These molecular comparisons will allow us to ensure that our metadynamics methods have properly ascertained the probability of embedded binding and allow us to determine if the physiochemical properties of these molecules are typical of membrane-embedding molecules.

From the set of compounds used for this work, compounds **1**-Az-H and **2**-St-H have carboxyl groups, whereas **4**-Z-Dy-Me and **5**-E-Dy-Me have a methyl ester. Carboxyl groups deprotonate in aqueous biological conditions (pH ∼7.4) to yield carboxylates, which are especially hydrophilic. However, inside a membrane, the less hydrophilic protonated states would likely predominate. Naïvely, this implies that both protonation states should be considered for these molecules. We chose to focus on the protonated states because they would less strongly bias the head groups to be hydrophilic, allowing the properties of the 2*H*-azirine moiety to more strongly determine the outcomes of our study.

## Computational Methods

### Force Fields

The CHARMM36 force field was used to model all molecular interactions.^34−37^ However, force fields for molecules containing the carboxy-2*H*-azirine moiety are unavailable in CHARMM. CGenFF can be used to automatically generate all of the parameters needed to run an MD simulation of a new small organic molecule.^25,38^ The parameters are determined by analogy to molecules already parametrized in the CHARMM force field and are assigned “penalties” rating the appropriateness of the analogy. A penalty larger than 50 indicates an extremely poor analogy and thus an unreliable parameter, which should be manually determined.^25^

**Figure 2:**
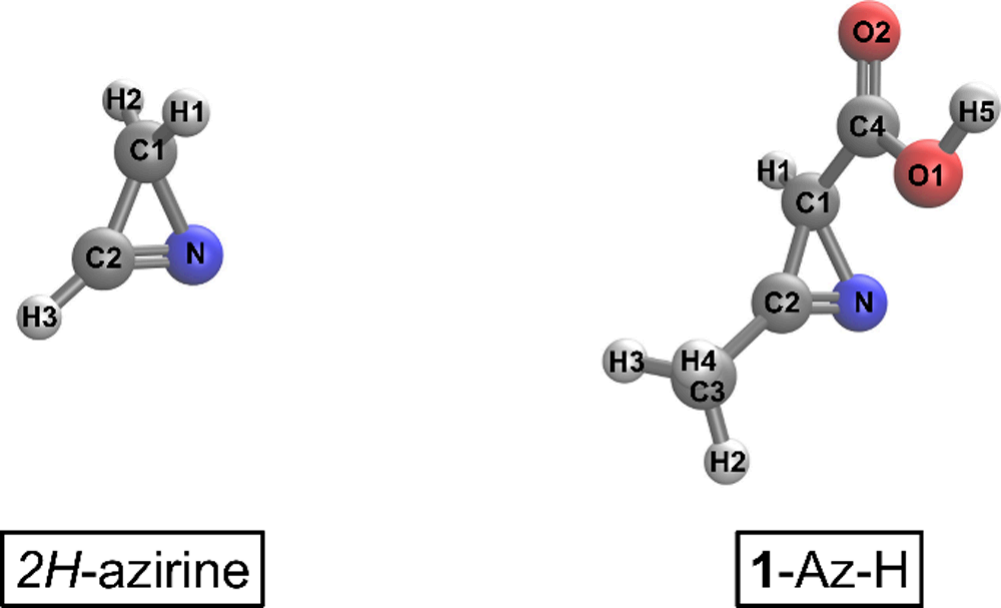
Ball and stick structures of the two central molecules used to develop our azirine force field. Carbon atoms are gray, nitrogen atoms are blue, hydrogen atoms are white, and oxygen atoms are red.

In order to obtain initial atomic point charges for the central azirine ring of the carboxy-2*H*-azirine moiety, we first focused on the simple 2*H*-azirine molecule (i.e., C_2_H_3_N). The structure of this molecule is shown in Figure 2. First, we used CGenFF to obtain initial force field parameters. The charge for the C2 atom had a low penalty, but the N and C1 charges required manual parametrization. To parametrize charges which are compatible with the CHARMM force field, one must fit interactions with TIP3P water to quantum chemical potential energy surfaces for interactions with water.^39^ To perform these calculations, we used the program FFParam.^39^ This program was used to optimize the N and C1 atomic charges by fitting them to quantum mechanical water interaction energies (MP2/6-31G*) computed using Psi4 1.5.^36,40−42^ As a check, the fitted charges were compared to a CHELPG charge analysis of 2*H*-azirine in Q-Chem 5.4 at MP2/6-31G*, and we found that both sets of charges were similar. This includes the C1 atom, which was not changed from the CGenFF guess.^43,44^

Next, we parametrized **1**-Az-H as our minimal example of a carboxy-2*H*-azirine-containing molecule. This structure is also shown in Figure 2. After using CGenFF to obtain the initial force field, we observed that many of the high penalty interactions included C1. Using constrained optimizations in Psi4 1.4 (for bonds and angles) and Q-Chem 5.4 (for dihedrals), we obtained potential energy surfaces for all high penalty (i.e., >50) interactions except for dihedrals involving hydrogen, which FFParam does not recommend parametrizing.^39,42,44^ These potential energy surfaces were obtained at the MP2/6-31G* level of electronic structure theory, and all degrees of freedom besides the one being scanned were optimized at each step. All degrees of freedom were scanned starting from the optimized geometry. Bonds were scanned in 17 steps from 15 pm less than the optimized length to 15 pm more than the optimized length, and the optimized length was the central ninth step. In a similar way, angles were scanned in 11 steps from 15° below to 15° above the optimum angle. For the dihedrals, full scans from −160° to + 160° were attempted. In several cases, only a portion of the potential energy surface could be collected due to high ring strain or interatomic clashes during optimization. However, the portion of the potential energy surface collected was always sufficient to fit the interaction of interest; the probability of the molecule exploring the unscanned coordinate values in an ordinary 300 K MD simulation was negligible. Dihedrals were fit to the appropriate CHARMM sum-of-cosines functions, carefully monitoring how few could be used to achieve a satisfactory fit of the potential energy surface. One out-of-plane improper dihedral angle was scanned from −5° to 5° and fit to a harmonic interaction.

The charges for **1**-Az-H were obtained in the following way. First, CGenFF was used to obtain initial charges. A CHELPG analysis (MP2/6-31G*) was used to help constrain the charge optimization space. Comparing the CGenFF penalties, the CHELPG analyses, and the charges obtained for 2*H*-azirine, we believed that the charges for N and C1 would be similar between the two molecules. If the charges from 2*H*-azirine for the analogous atoms are used for N and C1 in **1**-Az-H and the CGenFF values are used for all other charges, then the overall charge is −0.209. According to CHELPG, C2 in **1**-Az-H would be expected to become more positive than C2 in 2*H*-azirine, and CGenFF agrees with a penalty less than 50. The CGenFF penalty for C4, however, was greater than 50, and the CHELPG analysis implied that it should be more positive than CGenFF suggested. Thus, +0.209 atomic units of charge were added to this atom. This leaves the overall molecule neutral. The final obtained point charges correlate well with synthetic observations of the electrophilicity and nucleophilicity of the atoms in 2*H-*azirine-bearing molecules.^45,46^ The N and C1 are predicted to be nucleophilic, and C2 is predicted to be electrophilic.

The high penalty interactions for **4**-Z-Dy-Me and **5**-E-Dy-Me (which are geometric isomers and so have the same force field parameters) were the same as for **1**-Az-H, so the required force field parameters were simply borrowed. Outside of the carboxy-2*H*-azirine group, all CGenFF penalties were small for **4**-Z-Dy-Me and **5**-E-Dy-Me. The initial (CGenFF) and final (FFParam) values of all the force field parameters and the CHELPG analyses are given in the SI. Some examples of how the coordinates of interest were scanned and fit are provided on github at https://github.com/Daly-Lab-at-Haverford/code_examples. Similar strategies to those described in this section have been used to obtain CHARMM-compatible parameters for a wide variety of molecules.^47−52^

### Molecular Dynamics Systems and Equilibration

**Figure 3:**
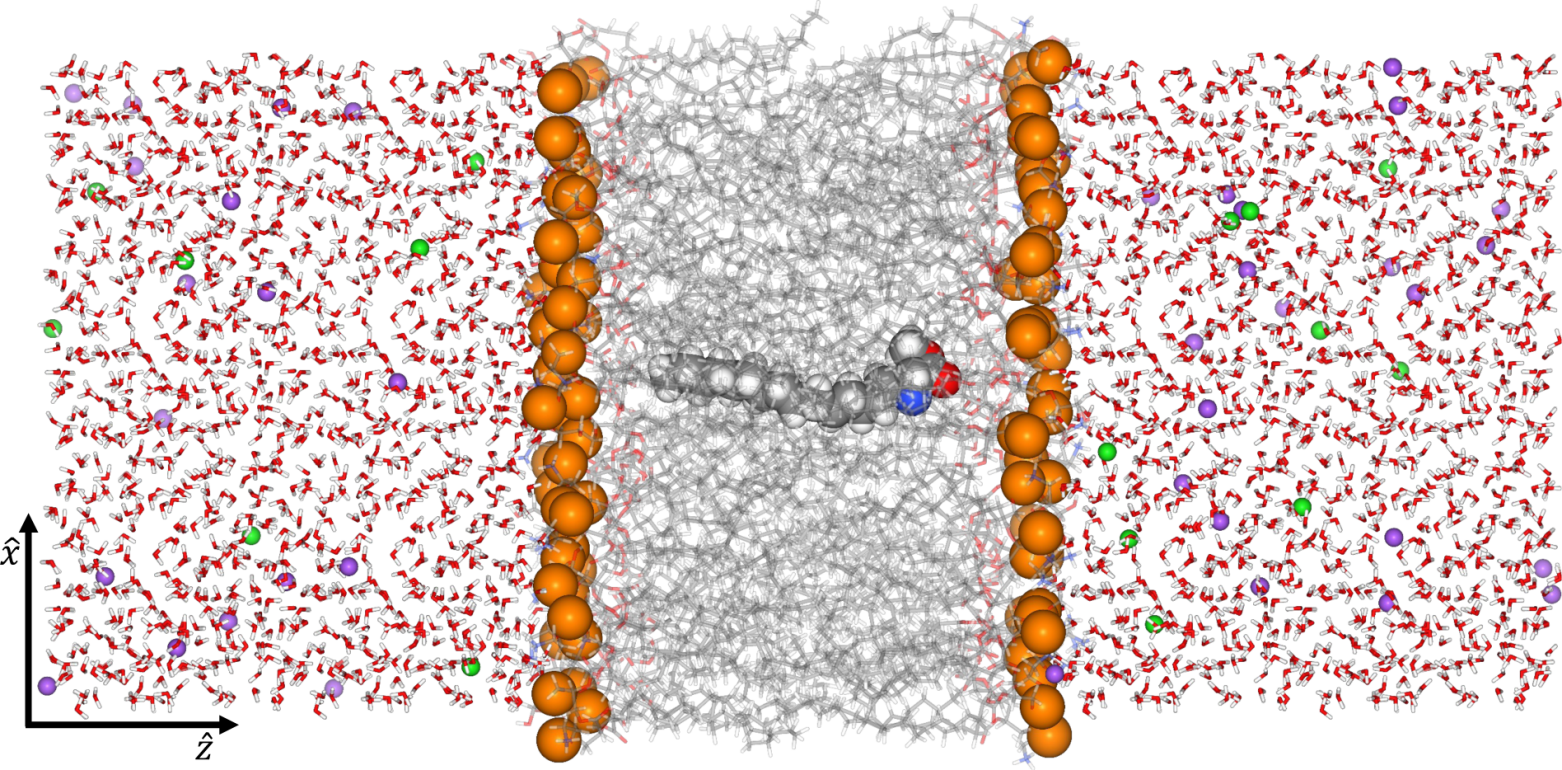
Initial structure of **5**-E-Dy-Me in the membrane prior to equilibration. Water molecules (red and white) are shown in a licorice representation, and NaCl (purple and green, respectively) ions are shown as small spheres. Licorice POPE and POPG molecules (gray, white, red, and blue depending on the atom type) are slightly transparent to more easily show **2**-E-Dy-Me, which is shown in a space filling representation. Phosphorus atoms (orange) are also shown in a space filling representation to clearly demark the boundary between the hydrophilic and hydrophobic portions of the simulation. On the bottom left, the directions of the *x̂* and *ẑ* unit vectors are shown. The *ŷ* unit vector is perpendicular to both of these and so points toward the reader by the right-hand rule. The correspondence between colors and elements is: blue for nitrogen, red for oxygen, white for hydrogen, green for chlorine, purple for sodium, orange for phosphorus, and gray for carbon.

Initial structures of our small molecules embedded in a membrane were constructed using CHARMM-GUI’s membrane builder.^53−56^ The membrane bilayer was constructed from 75% palmitoyloleoyl phosphatidylethanolamine (POPE) and 25% palmitoyloleoyl phosphatidylglycerol (POPG) and was oriented so that its surface plane was aligned with the *xy* plane. This mixture has previously been used as an effective model of the inner bacterial membrane.^57^ The *x*- and *y*-axis lengths of the membrane and the periodic box were initially 5 nm. Above and below the membrane, 4 nm of water was added; NaCl was added to neutralize the membrane and then to a total ionic strength of 0.15 M. The structure of each solute was initially obtained using Avogadro.^58^ A distinct system was created for each solute where the solute was placed in the center of the membrane. CHARMM-GUI was allowed to perform some minimization of the energy of the structure. An example initial membrane structure with a solute inserted is shown in Figure 3.

The membrane systems taken from CHARMM-GUI were subjected to the equilibration procedure suggested by CHARMM-GUI, which was implemented in OpenMM 7.6.^59^ The steps of this equilibration procedure have been outlined by Jo et al., though in the most recent version, the *NP*_z_γ*T* (called NPAT in their work) simulation steps are each 500 ps.^54,60^ To produce NP_z_γT ensemble simulations, we held the surface tension (γ) at 0 kJ/mol/nm^2^, the pressure in the *z*-axis direction (*P_z_*) at 1 bar, and the temperature (*T*) at 300 K.^61^ A Monte Carlo barostat with an update frequency of 150 steps was used to maintain the pressure and surface tension.^62^ Temperatures were controlled using Langevin dynamics with a friction coefficient of 1 ps^–1^.^63^ All simulations were performed in this ensemble. Nonbonded interactions were cut off after 1.2 nm with a smooth switching function used from 1.0 nm. Long-range electrostatics were corrected using particle mesh Ewald summation with an error tolerance of 0.0005. Configurations were sampled every 10 ps. After the equilibration recommended by CHARMM-GUI, velocities were randomized according to the Maxwell–Boltzmann distribution at 300 K, and the system was equilibrated with no constraints for 2 ns.

### Well-Tempered Metadynamics

PMFs were obtained using an OpenMM implementation of well-tempered metadynamics.^32^ In this method, biases are added to the system along a CV of interest, encouraging the system to explore the full range of values of the CV. As specific values of the CV are sampled, the strength of the biases added at those CV values is reduced. At long times (i.e., *t* → ∞), the added biases will converge such that the bias along the CV stops changing. The total added bias is related to the PMF, *f*(*z*),
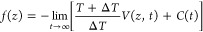
1where *z* is the CV, *T* is the temperature of the simulation (300 K in this case), the biases are added in such a way that the CV is sampled as if the temperature was *T* + Δ*T*, *V*(*z*, *t*) is the total added bias as a function of *z* and time, *t*, and *C*(*t*) is a constant, which can be ignored once convergence is reached.^32^ In our simulations, *T* + Δ*T* = 6000 K (i.e., the bias factor was 20). The CV was chosen to be the distance between the *z*-axis location of the center of mass of the molecule of interest and the *z*-axis location of the center of mass of all POPE and POPG molecules (i.e., the central plane of the membrane). The biases were shaped as Gaussian functions with a standard deviation of 0.3 nm. The initial bias height was 25 kJ/mol, and they were added every 100 fs. The CV was allowed to vary from −6 nm to +6 nm. All metadynamics simulations were run under *NP_z_*γ*T* conditions (1 bar, 0 kJ/mol/nm^2^, and 300 K). Similar CVs and metadynamics settings have been used in previous studies of membrane embedding and partitioning.^64−66^ Because of the symmetry of the system, the converged PMF should be even such that *f*(*z*) = *f*(−*z*). Metadynamics simulations were run for 2 μs and then continued in 500 ns increments until three criteria were met: (1) the RMSD between *f*(*z*) and *f*(−*z*) was at most 4 kJ/mol for any specific value of *z*, (2) the RMS standard error over all values of *z* between *f*(*z*) and *f*(−*z*) was below 2.5 kJ/mol, and (3) the RMSD between the potential of mean force obtained at the end of the simulation and that obtained 50 ns prior to the end was less than 2.5 kJ/mol across all values of *z*. This took different amounts of simulation time for each molecule (Table 1). Due to these criteria, the ones place is treated as the final significant place value in the free energy values obtained in this work.

**Table 1:** Time to Convergence for Each Metadynamics Simulation

**molecule**	**time (μs)**
**1**-Az-H	2.0
**2**-St-H	2.0
**3**-Sp	4.0
**4**-Z-Dy-Me	3.0
**5**-E-Dy-Me	2.0

### Membrane Partitioning

A major question in this study is the extent to which the molecules of interest embed in the membrane compared to dissolving into the water layer. This can be captured using the partition coefficient, defined as
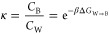
2where *C*_W_ and *C*_B_ are the concentrations of solute molecules in water (W) or in the bilayer (B), respectively, , and Δ*G*_W→B_ is the partitioning free energy describing moving a molecule from the water to the bilayer and is given by
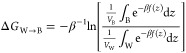
3where *V*_W_ and *V*_B_ are the volumes of the water and bilayer regions, respectively, and the integrals are taken over these regions.^67−69^ In this work, we defined the bilayer region to range from −3 < *z* < 3 and the water region to include all other space. While the bilayer region as defined includes some volume that may intuitively be considered part of the water region, we include this volume for two reasons. First, this definition makes *V*_W_ and *V*_B_ equal, simplifying our calculations and interpretations. Second, the bilayer has some influence on the space outside the phosphate head groups, and we believe that this should be included in our investigation of its interactions on the molecules of interest.

## Results

### Potentials of Mean Force

**Figure 4:**
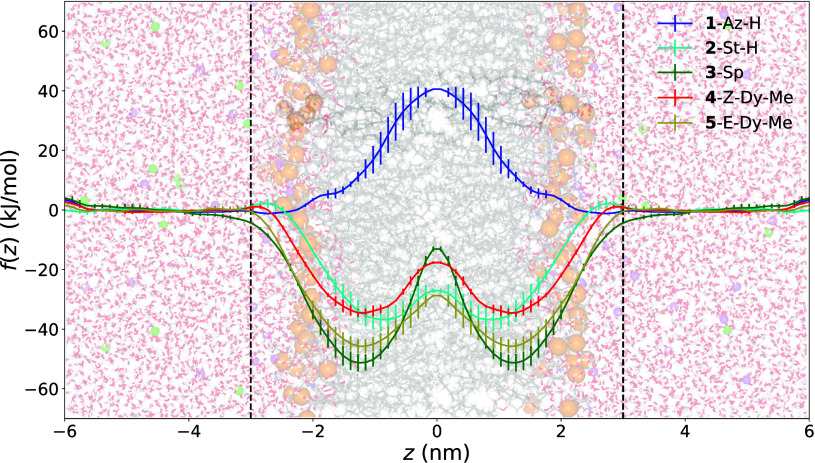
Potentials of mean force for the *z*-axis distance between the membrane center of mass and the center of mass of the molecule of interest. Values are averages between *f*(*z*) and *f*(−*z*), and error bars are 95% confidence intervals. For context, a simulation snapshot is shown behind the plots. The zero of energy in this plot is the average value in the water region (|*z*| ≥ 3). The boundaries between the two regions are shown as black dotted lines. The correspondence between colors and elements is: blue for nitrogen, red for oxygen, white for hydrogen, green for chlorine, purple for sodium, orange for phosphorus, and gray for carbon.

The PMFs (Figure 4) clearly show that **1**-Az-H is distinct from the other molecules. The small Δ*G*_W→B_ value and the κ value near unity (Table 2) show that **1**-Az-H is essentially indifferent to the choice between the bilayer and water regions. The long-chain molecules and **2**-St-H, **3**-Sp, **4**-Z-Dy-Me, and **5**-E-Dy-Me all strongly prefer to embed within the membrane over all other possibilities. There is a substantial difference between the binding strengths of **4**-Z-Dy-Me and **5**-E-Dy-Me; the *E* isomer is significantly more stable in the membrane. The partitioning values for **2**-St-H are fairly similar to those for **4**-Z-Dy-Me. Meanwhile, **3**-Sp is similar to **5**-*E*-Dy-Me.

**Table 2:** Free Energy Changes Obtained for the Partitioning of the Molecules to the Membrane in kJ/mol and Unitless Partition Coefficients^a^

**molecule**	**Δ***G*_**W→B**_	log κ
**1**-Az-H	2	0
**2**-St-H	–33	+6
**3**-Sp	–46	+8
**4**-Z-Dy-Me	–31	+5
**5**-E-Dy-Me	–42	+7

a Values are rounded to the nearest ones digit.

All four molecules have similar hydrophobic regions, but **3**-Sp has a highly hydrophilic head group, while the head group of **2**-St-H is more modestly hydrophilic. These trends can be explained if we suppose that the hydrophilicity of the carboxy-2*H*-azirine group is only a little weaker than that of the head group of **3**-Sp. For **3**-Sp and **5**-E-Dy-Me, moving from water to the bilayer region allows the molecules to begin to hide their hydrophobic tails in the membrane. Moving too far into the membrane, however, is unfavorable because of the hydrophilicity of their head groups. For **2**-St-H, the interfacial area at the start of the bilayer region does not provide sufficient opportunity to hide its hydrophobic tail, which is longer than for any of the other molecules. When embedded, it is able to move more deeply into the membrane because of the relatively weak hydrophilicity of its head group. **4**-Z-Dy-Me has less exposed hydrophilic and hydrophobic surface area than **5**-E-Dy-Me because of its shape. This weakens the penalty for remaining exposed to the liquid, making Δ*G*_W→B_ more positive for 4-Z-Dy-Me.

These partitioning free energies can be compared to partitioning free energies obtained in other membrane partitioning investigations. We also include comparisons to binding free energies or differences in the PMF between a water region and a membrane region since their values are often fairly similar to partitioning free energies in this context. The PMFs of membrane components partitioning into a wide variety of membranes were computed by Rogers et al. (for phospholipids) and Ermilova and Lyubartsev (for cholesterol). Free energy changes upon removal from the bilayer ranging from −32.8 to −72.4 kJ/mol were obtained, which broadly agreed with experiment.^64,70^ Other studies focused on the binding of hydrophobic molecules. MacCallum and Tieleman found a free energy change of −21 kJ/mol for incorporating hexane into a bilayer, in agreement with experiment (estimated at −24.8 kJ/mol), and Bochicchio et al. obtained a free energy change near −30 kJ/mol for small hydrocarbons.^66,69^ Johansson and Lindahl (for amino acid side chains) and Jämbeck and Lyubartsev (for small drug molecules) examined the membrane binding of broadly amphiphilic molecules. The partitioning depends sensitively on the specific characteristics of the molecule of interest. Observed free energy changes are as low as −22.2 kJ/mol, but binding is unfavorable for many of the polar or charged amino acids (the binding free energy was not calculated).^65,71^ Most of the free energies obtained in these studies agree well with experiment.

The PMF for **1**-Az-H obtained in this work is similar to those obtained for polar or charged amino acid side chains, having a large peak near *z* = 0. Interestingly, the peak in the PMF for **1**-Az-H is about 3 times as large as the largest observed by Johansson and Lindahl for their side chain analogue of aspartic acid, about 12 kJ/mol; this confirms that **1**-Az-H is strongly hydrophilic.^71^ Our Δ*G*_W→B_ values for **2**-St-H, **3**-Sp, **4**-Z-Dy-Me, and **5**-E-Dy-Me fit into the high (unfavorable) end of the range for membrane components but are lower (more favorable) than values for hydrophobic molecules or small drug molecules. Our long-chain molecules of interest are similar to the investigated membrane components studied by Rogers et al. but have smaller hydrophilic and hydrophobic regions, leading to generally weaker binding.^70^ The hydrophilic portions of our molecules of interest lead to stronger binding than completely hydrophobic molecules, and the long 18 carbon chains of our molecules of interest lead to stronger binding than small amphiphilic molecules. Overall, the partitioning free energies obtained in this work fall into a reasonable range compared to similar free energy calculations in the literature.

### Binding Probability Distributions and Orientation

The structural relationship between our molecules and the membrane is vitally important. It is not sufficient to know that a carboxy-2*H*-azirine-bearing molecule embeds within the membrane. We must also ask if the carboxy-2*H*-azirine group itself is available for further biological interactions. This question was addressed using the unbiased probability densities for all sampled configurations. After our simulations converged, we used the reweighting method of Branduardi et al. to weight the statistics of the simulation configurations observed under metadynamics.^72^ In this method, the unbiased probability, *P*_*u*_(***r***), of a given simulation configuration ***r*** (representing all atomic positions) is given by
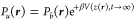
4where *z*(***r***) is the value of the CV in the configuration ***r*** of interest, *P*_*b*_(***r***) is the biased probability with which the configuration was observed under metadynamics, *V*(*z*(***r***), *t*→∞) is the converged bias surface, and .^72,73^ The quantity e^β*V*(*z*(***r***), *t*→∞)^ can be thought of as a weighting factor, which removes the effect of the bias potential, leaving only the “true” free energy surface to affect configurational probabilities. However, using metadynamics has allowed for the sampling of configurations across *z* even if their unbiased probability was marginal, allowing for more complete statistics.

**Figure 5:**
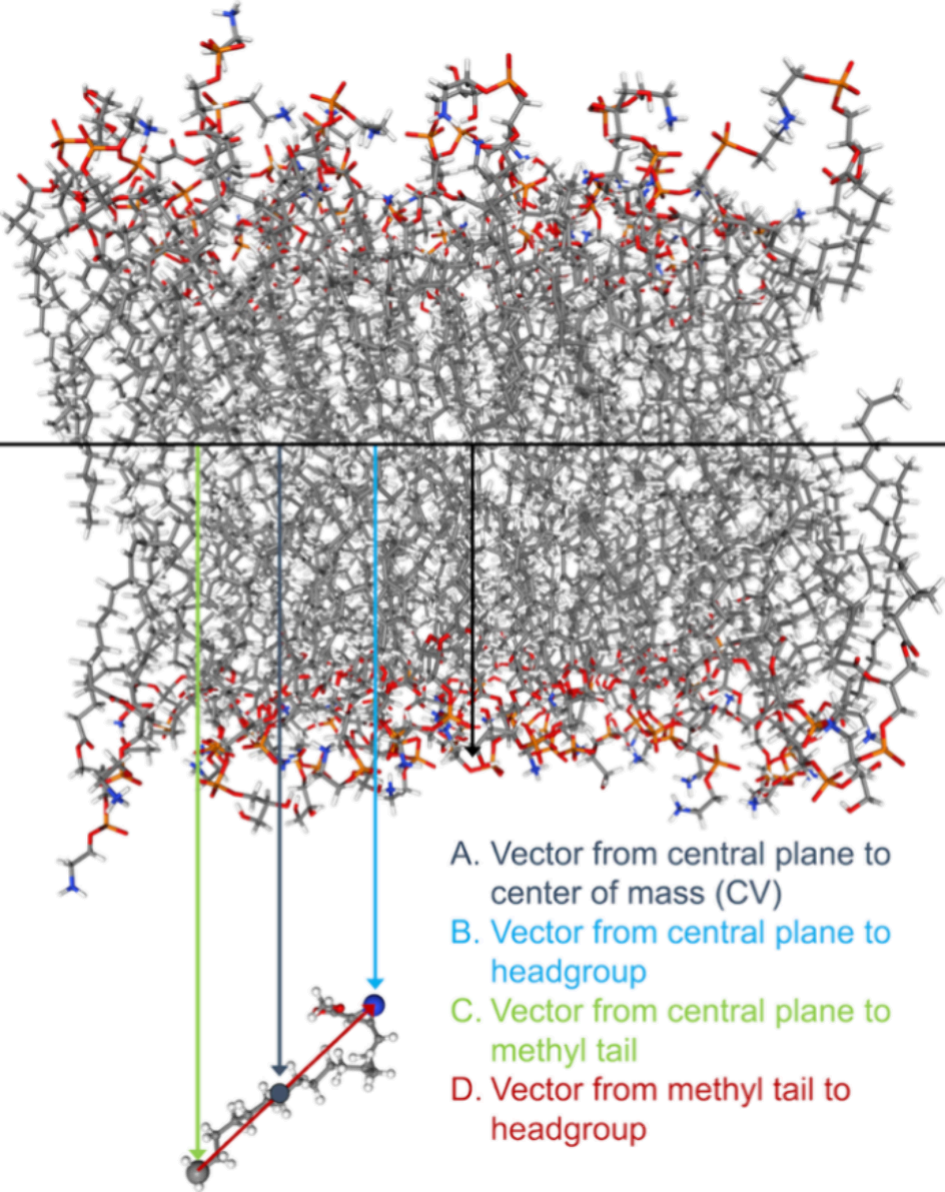
Vectors used to define the unbiased probability distributions shown in Figure 6. The pictured molecule of interest is **5**-E-Dy-Me. Water and ions have been removed for clarity. Per the results shown below in Figure 6, the pictured configuration is highly unlikely in the real system. The correspondence between colors and elements is: blue for nitrogen, red for oxygen, white for hydrogen, orange for phosphorus, and gray for carbon.

**Figure 6:**
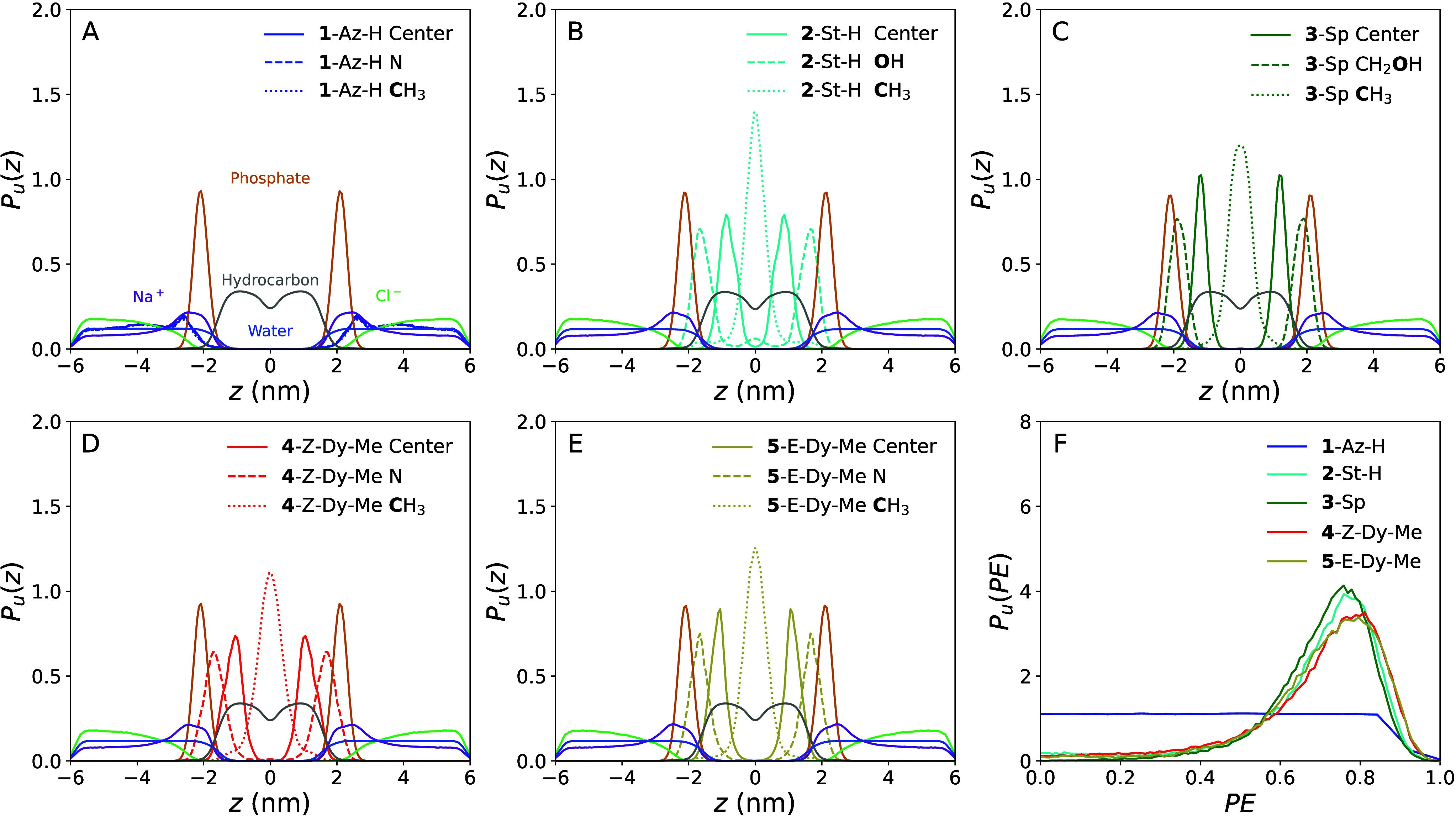
(A–E) Unbiased probability density for the *z*-axis distance for several of the constituents of each simulation: the water oxygens, the hydrophobic carbons in the membrane, the NaCl ions, and the head groups, tail groups, and centers of mass of the molecules of interest. Part A includes color coded labels for the nonsolute portions of the simulations; the same colors are used in all parts of the figure. Each plot contains data from simulations of a different solute: (A) **1**-Az-H, (B) **2**-St-H, (C) **3**-Sp, (D) **4**-Z-Dy-Me, and (E) **5**-E-Dy-Me. (F) Parallel elongation of the solutes compared to the membrane surface normal, PE.

For each molecule, orientational information was obtained by tracking the locations of their centers of mass, head groups, and tail groups. For **1**-Az-H, **5**-E-Dy-Me, and **4**-Z-Dy-Me, the head group was represented by the nitrogen atom. For **3**-Sp and **2**-St-H, an oxygen atom was selected. For all our molecules of interest, the carbon of the methyl at the end of the carbon chain was selected to represent the tail end of the molecule. A depiction of these locations is shown in Figure 5. The locations of water oxygens, NaCl ions, hydrophobic membrane carbons, and phosphorus atoms were also tracked. The unbiased probability distributions of these atoms are shown as a function of the distance from the membrane central plane, *z*, in Figure 6A–F. In this figure, all distributions are normalized such that the integrated total probability is 1.0.

The behavior of the membrane, water, and ions is essentially identical across simulations of each solute. Hydrophobic carbons are strongly populated near the center of the membrane. The phosphorus atoms peak at the interface between the hydrophobic and hydrophilic portions of the simulation. Because the phosphate groups are strongly negative and the POPG molecules have a net negative charge, there is a peak in the distribution of Na^+^ ions overlapping with the phosphorus atom distribution. For similar reasons, the Cl^–^ ion distribution is peaked far from the membrane. These can be compared to the water molecule distribution, which is flat essentially everywhere outside the hydrophobic region of the membrane. If the membrane was not present, then we would expect the distribution of water molecules to be completely flat and identical to the distribution of NaCl ions. The water and NaCl ion distributions show decreased probability near |*z*| = 6 nm. This likely arises from the constant pressure control in the *z*-dimension; there were many configurations where the box was slightly smaller than 12 nm, so distances near ±6 nm were unphysically less likely to be observed. This has no effect on the major results of this work.

As expected from the PMFs shown in Figure 4, the center of mass of **1**-Az-H is preferentially located outside the membrane (Figure 6**A**). There are two peaks in its probability distribution: The largest occurs just outside the phosphorus peak, indicating favorable interfacial interactions. The smaller peak occurs just as the Cl^–^ ions start to become more probable than the baseline set by water molecules. Tracking the nitrogen and methyl carbon of **1**-Az-H allows us to extract orientational information about the molecule. The distributions for these atoms are nearly identical to that for the center of mass. This can only be true if there is no orientational preference for **1**-Az-H. Focusing on the largest peak near the phosphate groups, the peak for the nitrogen at |*z*| = 2.56 nm is very slightly closer to the phosphate groups than the peak for the methyl carbon at |*z*| = 2.62 nm. There are three atoms in **1**-Az-H with substantial positive charges, which might be expected to interact favorably with phosphates: H5, C4, and C2 (Figure 2). Considering the geometry of **1**-Az-H, it might be that the carboxy group is engaging in hydrogen bonds with phosphates. However, even this orientational preference is slight. Without a long hydrocarbon tail, **1**-Az-H is hydrophilic.

The distributions for **4**-Z-Dy-Me and **5**-E-Dy-Me are similar (Figure 6D,E). For both, the most probable location for the center of mass is near |*z*| = 1 nm. The head group is located near the phosphate groups. In fact, the phosphorus and nitrogen distributions overlap substantially. However, the nitrogen atoms are unlikely to be located beyond the phosphorus atoms. Moving out of the membrane, the head group probability goes to zero before the phosphate probability does. The methyl tails are consistently located at the center of the membrane. All the probability distributions for **5**-E-Dy-Me are somewhat sharper than the equivalents for **4**-Z-Dy-Me, as should be expected from the relative Δ*G*_W→B_ values for the two molecules. Together, we see that **4**-Z-Dy-Me and **5**-E-Dy-Me are broadly aligned with the membrane lipid molecules with their head groups located at the start of the hydrophilic region. This strongly implies that their head groups are hydrophilic. Together with the results from **1**-Az-H, we can conclude that the carboxy-2*H*-azirine moiety is hydrophilic.

The two remaining molecules, **3**-Sp and **2**-St-H, have similar distributions but with important differences (Figure 6B,C). Both are aligned with the membrane and have their head groups pointed toward the hydrophilic region. However, the head group of **3**-Sp overlaps more than any other molecule of interest with the membrane phosphorus atoms. For this molecule, we also see that the tail group probability distribution is relatively broad. The center of mass peak is relatively sharp for **3**-Sp, which corresponds well with the especially negative Δ*G*_W→B_ observed for that solute. This is all consistent with the idea that the head group of **3**-Sp is highly hydrophilic and is more hydrophilic than any other membrane-embedding molecule of interest. The head group distribution for **2**-St-H overlaps the least with the phosphorus atom distribution. In fact, there is a small peak in the head group distribution at the center of the membrane. The tail group distribution is more strongly peaked at the center of the membrane than any other molecule of interest; at the same time, there is a small probability that the tail group is located near the phosphate groups. The center of mass of **2**-St-H is consistently located between the head and tail groups. This means that in most cases, the molecule is aligned with the membrane phospholipids; however, **2**-St-H is also occasionally antialigned with the membrane. This is consistent with the idea that the head group of **2**-St-H is more hydrophobic than the head group of any other molecule of interest. Together, **3**-Sp and **2**-St-H can be seen as behavioral end points that **4**-Z-Dy-Me and **5**-E-Dy-Me fall between.

The above results already strongly imply that the molecules **2**-St-H, **3**-Sp, **4**-Z-Dy-Me, and **5**-E-Dy-Me are most often embedded in the membrane and oriented with their head group pointed toward the solvent and with their methyl tails pointed into the center of the membrane, a strong orientational preference. To confirm this, we computed a direct measure of alignment with the membrane bilayer. We assumed that the membrane surface normal unit vector was always identical to the *z*-axis unit vector, *ẑ* . Then, we defined the parallel elongation of the solutes with the membrane, PE, as
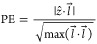
5where  is the vector pointing from the methyl tail to the head group atom in a particular molecule. The denominator of eq 5 is the maximum molecule length ever observed in our simulations. Our metadynamics simulations sample a wide variety of molecular configurations, so this is a slight overestimate for the length of each molecule at “full” elongation. The numerator is the length of the molecule in a specific configuration, but only in the *z*-axis direction. When PE = 1, the molecule is fully elongated and is perfectly parallel to the *z*-axis. In other words, PE = 1 describes perfect alignment with a single leaflet of the membrane. The PE = 0 end point can describe several possibilities. For instance, the molecule could be completely elongated but could have a 90° angle with respect to the membrane surface normal. Alternatively, PE will be close to zero if the molecule has a small radius of gyration. In any case, values of PE near zero describe a distinct lack of alignment with the membrane. The unbiased probability distribution of alignment values observed in our simulations is given in Figure 6F. The results clearly show that all molecules of interest are most often parallel to the normal vector and elongated except **1**-Az-H. All PE values for **1**-Az-H are equally probable, as would be expected from the results shown in Figure 6A. The probability of PE values especially close to 1 is lower than might be expected because the maximum molecular lengths used to define perfect elongation require longer than equilibrium bond lengths, which are generally energetically unfavorable.

## Discussion

This work was performed with compounds **1**-Az-H, **4**-Z-Dy-Me, and **5**-E-Dy-Me as representatives of the natural product carboxy-2*H*-azirine set. We selected **4**-Z-Dy-Me and **5**-E-Dy-Me because these compounds are geometric isomers, and such structural differences are known to impact membrane-dependent cell functions by altering membrane fluidity.^74,75^ Thus, membrane embedding may be expected to differ between these two isomers, possibly leading to distinct mechanisms of inhibition. Comparing the linear alkyl compounds composed of an 18-carbon chain, **2**-St-H, **3**-Sp, **4**-Z-Dy-Me, and **5**-E-Dy-Me, against a short-alkyl-chain compound composed of a 4-carbon chain, **1**-Az-H, allowed us to investigate the effect of carbon chain length on membrane embedding. Long alkyl chains in small-molecule inhibitors can be distinct drivers of membrane integration,^33,76^ but the alkyl chain length and chain saturation can also influence binding affinity with a membrane-bound molecular target.^77,78^ We also sought to identify any perturbations that the carboxy-2*H*-azirine could have on the ability of long-chain molecules to embed in a membrane. Many of these properties depend sensitively on the hydrophilicity or hydrophobicity of the carboxy-2*H*-azirine group.

Our results strongly predict that, at equilibrium, molecules **2**-St-H, **3**-Sp, **4**-Z-Dy-Me, and **5**-E-Dy-Me will display embedded binding to a lipid membrane, while **1**-Az-H will not. Even so, **1**-Az-H can display interfacial binding (Figure 6A). For the other molecules in our set, moving from the water to the membrane is always favorable. For molecules **2**-St-H, **3**-Sp, **4**-Z-Dy-Me, and **5**-E-Dy-Me, the reverse process of moving from the membrane to water is highly unfavorable and essentially will never be observed in experiment. The large free energy changes observed in this study give us the confidence to predict that partitioning to the membrane will remain favorable for molecules **2**-St-H, **3**-Sp, **4**-Z-Dy-Me, and **5**-E-Dy-Me even if the composition of the membrane is changed substantially from our particular mixture of 75% POPE and 25% POPG.

We also predict that the carboxy*-*2*H-*azirine moiety is sufficiently hydrophilic to act in much the same way as the amino and carboxy groups present in **3**-Sp and **2**-St-H, respectively. The charge distribution in our force field, supported by quantum chemical CHELPG population analysis, is consistent with other hydrophilic compounds. For instance, the charges adopted for the N (−0.357) and the C2 (+0.427) in the 2*H-*azirine ring are similar in magnitude to those of the H atoms in TIP3P water (+0.417). We also find that **1**-Az-H, the smallest carboxy-2*H-*azirine natural product, strongly prefers aqueous solution to the hydrophobic space within the membrane. The carboxy*-*2*H-*azirine groups in **5**-E-Dy-Me and **4**-Z-Dy-Me also prefer aqueous solution, causing these molecules to orient with those moieties facing the water layer and to overlap substantially with the phosphate head groups in the membrane. This behavior is similar to that observed for **3**-Sp and **2**-St-H. Like these nonazirine-containing compounds, the hydrophobic tails of **5**-E-Dy-Me and **4**-Z-Dy-Me point toward the center of the membrane, and the molecules align with the membrane.

## Conclusions

In this work, we have made predictions of the membrane partitioning of carboxy-2*H*-azirine-containing molecules and determined that the moiety itself is likely hydrophilic, similar to an amino or hydroxyl group. Substantial membrane complexities encompassing phospholipids, cholesterol, proteins, and carbohydrates that give the membrane a fluid character could hypothetically be added to our model and may exist in the real systems.^79^ However, even in our simplified representation, we see the same behavior between molecules that are known to embed in complex membranes (**3**-Sp and **2**-St-H) and long-chain carboxy-2*H*-azirines (**5**-E-Dy-Me and **4**-Z-Dy-Me). This and the large free energy changes upon embedding that we observe allow us to speculate that similar partitioning would be observed if long-chain carboxy-2*H*-azirines were introduced to membranes containing substantial complexity, and we welcome future simulations and experiments testing this speculation.

Like other long-chain molecules with a hydrophilic head group, the mixture of polar and nonpolar interactions leads to long-chain molecules embedding in lipid bilayers with the hydrophilic carboxy-2*H*-azirine group colocated with the polar phospholipid head groups of the membrane bilayer. Gaining this understanding of the physiochemical properties of carboxy-2*H*-azirines utilizing metadynamics has been a crucial first step for guiding the elucidation of the biological mechanisms of inhibition from these molecules. Currently, biochemical experimentation using these molecules is hampered by the synthetic feasibility of generating enough material for comprehensive microbial assays with the complete panel of carboxy-2*H*-azirine natural products. This computational work allows us to make tentative predictions about potential biological targets for carboxy-2*H*-azirine inhibition based on membrane-embedding behaviors, hopefully guiding additional experiments on these molecules. Moreover, such an understanding opens up this unexplored chemical space, offering a distinct scaffold with promising potential for therapeutic development.

It is clear from our metadynamics simulations that **1**-Az-H is an expected outlier and must be separately investigated. Lacking a long alkyl chain, **1**-Az-H demonstrates complete aqueous partitioning due to the isolated hydrophilicity with the minimal carboxy-2*H*-azirine scaffold. This mode of binding suggests a distinct mechanism of action compared to those with longer alkyl chains; for instance, **1**-Az-H may target a soluble or a membrane embedded process. In contrast, the membrane partitioning for **4**-Z-Dy-Me and **5**-E-Dy-Me suggests that the whole set of long-alkyl-chain carboxy-2*H*-azirine analogs may target biological processes at, or near, the lipid membrane.^80^ This work has directed our first steps toward a membrane-centric investigation for uncovering the biological targets of the carboxy-2*H*-azirine natural products.

## Supporting Information Available

Additional details confirming wide configurational sampling in our metadynamics simulations, the initial CGenFF force field files for all of the small molecules, Q-Chem output files containing CHELPG analyses of 2*H*-azirine and **6**-Az-H, and the final force field files obtained using FFParam (ZIP)
(Figures S1–S4) Distribution of *X* and *Y* axis values sampled for the center of mass of each solute, orientational distributions of solute molecules outside the bilayer, and asymmetry of unbiased orientations (PDF)
