# Supplementary material for: Employing Metadynamics to Predict the Membrane Partitioning of Carboxy-2H-Azirine Natural Products
Source: J Phys Chem B. 2024 Sep 3;128(36):8771–81. doi: 10.1021/acs.jpcb.4c03411 (PMC11403667; doi:10.1021/acs.jpcb.4c03411)
Supplement: Supplementary file 2 — jp4c03411_si_002.pdf [file jp4c03411_si_002.pdf]

Supporting Information for: Employing Metadynamics to Predict the Membrane Partitioning of Carboxy-2*H*-Azirine Natural Products

Authors

Clyde A. Daly Jr.*[,1] (ORCID 0000-0003-2051-0561), Leah M. Seebald[1] (ORCID 0000-0001-8890-8985) and Emma Wolk[1]

[1] Haverford College
Department of Chemistry
370 Lancaster Ave
Haverford, PA 19041
United States of America

*Corresponding author, Phone: 610-896-1000, cdaly2@haverford.edu

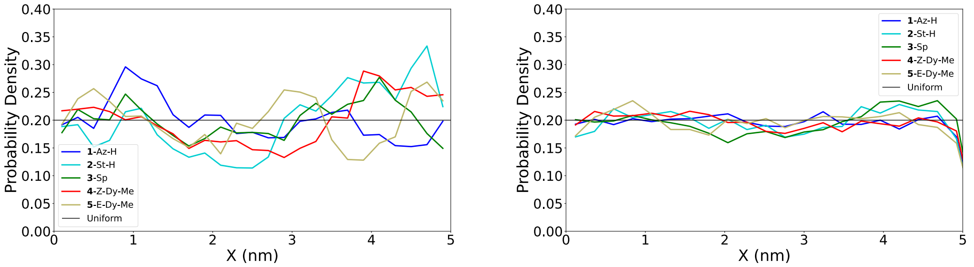

Figure S1: Distribution of X axis values sampled for the center of mass of each solute when inside (left) or outside (right) the bilayer.

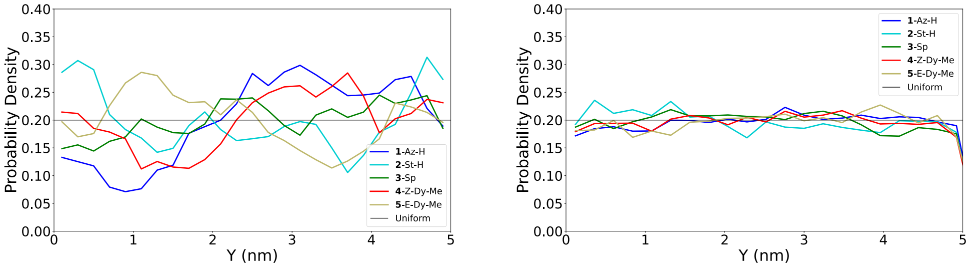

Figure S2: Distribution of Y axis values sampled for the center of mass of each solute when inside (left) or outside (right) the bilayer.

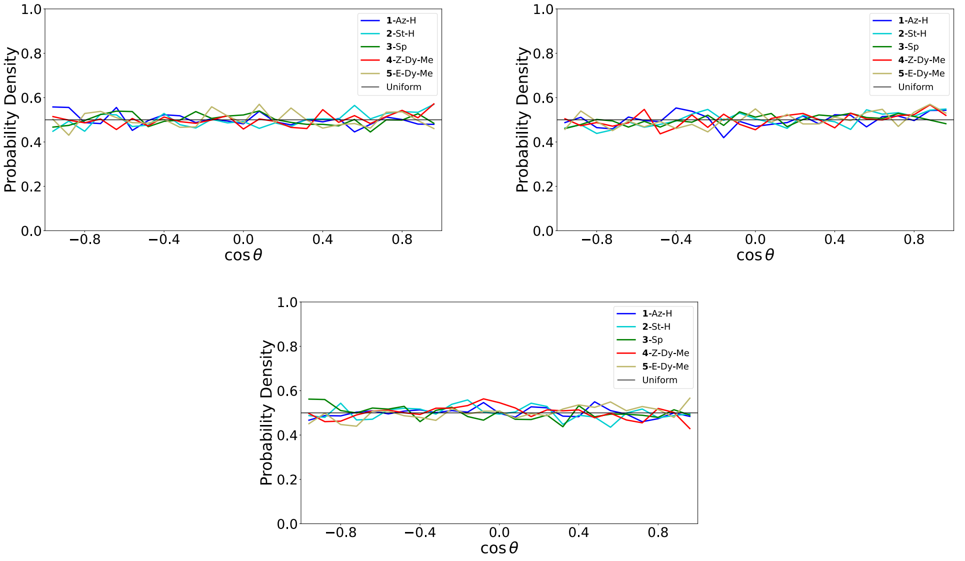

Figure S3: Orientational distributions of solute molecules outside the bilayer, plotted as the cosine of the angle between the dimensional unit vector and the molecular direction unit vector. (Top left) Comparison with X axis. (Top right) Comparison with Y axis. (Middle bottom) Comparison with Z axis.

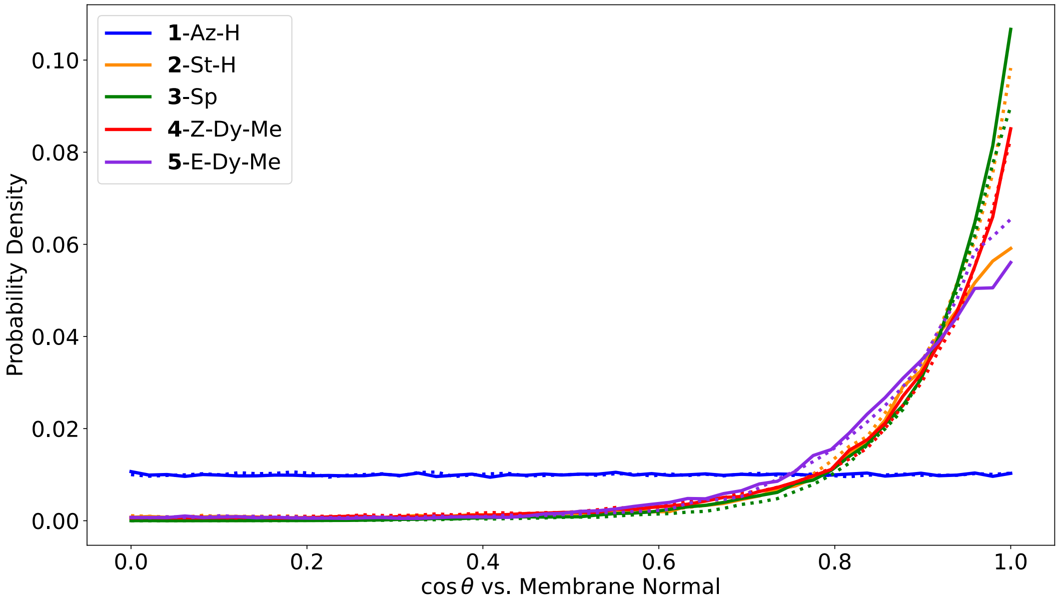

Figure 4: Asymmetry of unbiased orientations. Dotted lines are negative cosine values.
